# Spirulina ingestion and autoimmune disease onset or flare

**DOI:** 10.1186/s42358-025-00446-7

**Published:** 2025-03-15

**Authors:** Jozélio Freire de Carvalho, Ana Tereza Amoedo Martinez

**Affiliations:** 1 Núcleo de Pesquisa em Doenças Crônicas não Transmissíveis (NUPEC), School of Nutrition From the Federal University of Bahia, Salvador, Bahia Brazil; 2 Universidade Estadual de Feira de Santana, Feira de Santana, Bahia Brazil; 3 Novaclin, Grupo CITA, Salvador, Bahia Brazil

**Keywords:** Spirulina, Algae, Toxicity, Autoimmune diseases, Autoimmunity, Pemphigus, Dermatomyositis

Dear Editor,

Spirulina is widely utilized as a feed additive in agriculture and as a nutritional supplement for human consumption, owing to its rich concentration of proteins, vitamins, and minerals. Research suggests that Spirulina may have immunomodulatory effects. For instance, in vitro studies conducted on cat macrophages have shown that Spirulina can enhance phagocytic activity against *Escherichia coli* and sheep red blood cells, with minimal cytotoxic effects [1]. Moreover, an in vivo study involving chicks fed with Spirulina-enriched feed demonstrated an increase in the number of macrophages, leading to enhanced phagocytic activity and increased nitrite production in response to both lipopolysaccharide (LPS) and non-LPS stimuli [2]. Additionally, a study on humans revealed that oral administration of a hot water extract of Spirulina improved natural killer (NK) cell function, as evidenced by increased interferon production and cytolysis [3]. However, there is also concern regarding the potential of Spirulina to trigger autoimmune diseases. This review aims to examine the relationship between Spirulina ingestion and autoimmune diseases onset or flare. The literature on this field was screened until July 2024. No language restrictions were applied. Pubmed, Scopus, and Web of Science were the database analyzed.

The search found four articles with five cases reported [4–7]. The age varied from 45 to 82 years old, and 4/5 were female gender. Spirulina started dermatomyositis in 3/5 cases [4, 5, 7] and pemphigus in 2/5 [6, 7]. The time between spirulina ingestion and autoimmune onset varied from 1 to 2 days to 1 year. All patients with dermatomyositis had skin lesions compatible with this disease and proximal muscle weakness. The skin biopsy showed interface dermatitis in all cases. All these cases had high levels of creatine kinase, and aldolase was high in 2/3 cases (in the third case, this enzyme was not described). Creatine kinase values varied from 1268 to 4761 U/L and aldolase from 22.6 to 31.1 U/L. One patient had interstitial lung diseases and positive anti-Mi-1 antibodies [4]. One of the pemphigus cases developed a mixture of pemphigus foliaceous and bullous pemphigoid [7]; the other patient had a flare of previous pemphigus [6].

All patients had Spirulina suspended, and all cases received prednisone. Methylprednisolone was done in 2/5 cases (all dermatomyositis) [4, 5], intravenous immunoglobulin in 1/5 case [4], and cyclophosphamide in 1/5 [5]. One patient received hydroxychloroquine, topical tacrolimus, and cetirizine [7]. In 3/5 cases, the diseases improved or were cured after spirulina withdrawal and/or immunosuppressive drugs [4–6]. In 2/5, the outcome was not described in the article [7]. See Table 1 for a summary of all patient data.

**Table 1:** Studies on spirulin ingestion and autoimmune disease onset or flare

Author, year, reference	Study design	*N*, age, gender	Disease	Spirulina dosage	Time to disease onset	Clinical features	Treatment	Outcome
Kuzumi et al. 2024 [4]	Case report	1, 60 yo, Female	Anti-Mi-2-positive dermatomyositis	Unknown	1 month	Pruritus rash after 1 month of Spirulina ingestion 3 weeks after proximal muscle weakness and dysphagia Periungual erythema and nailfold bleeding CK of 4761 U/L and aldolase 31.1 U/L. Positive anti-Mi-1 antibody Electromyography-myopathy. Thorac CT: mild interstitiopathy Skin biopsy: interface dermatitis and perivascular infiltration, mucin deposition	Methylprednisolone pulse therapy -> 40 mg/day of prednisolone Monthly IVIg	Improved
Konno et al. 2011 [5]	Case report	1, 49 yo, Female	Myositis (probably dermatomyositis)	ND	Days	After taking Spirulina, she had a skin rash. After 5 months, she developed muscle weakness in her of the neck flexor and left proximal upper extremity. CK of 1,268 IU/ml Muscle biopsy: Many necrotizing muscle fibers, infiltration of mononuclear cells in the peri- and endomysium, including many eosinophils. Immunohistochemical: CD4-positive cells in the peri- and endomysium and CD20-positive B cells in the perivascular regions.	Prednisolone Cyclophosphamide Methylprednisolone pulse therapy	Improved
Kraigher et al. 2008 [6]	Case series	1, 82 yo, Female	Bullous pemphigoid and pemphigus foliaceus	ND	1 year	Bullae, partly hemorrhagic, are present on the trunk and extremities, secreting erosions and submammary macerations. Positive Nikolsky. 1st biopsy: subepidermal bulla with a denuded surface and sparse perivascular lymphocytic infiltrate with scattered eosinophils. 2nd biopsy: intra- and subcorneal vesicular dermatitis with slight superficial acantholysis. Direct immunofluorescence: IgG and C3 at the dermoepidermal junction. Indirect immunofluorescence: positive at the dermo-epidermal junction. Salt split test: IgG, IgM, and C3 on the upper side of the bulla. No pemphigus autoantibodies.	Prednisone 60 mg/day and spirulin withdrawal.	She was cured after 3 months.
Lee & Werth, 2004 [7]	Case series	2 out of 3, Case 1: 57 yo male Case 2: 45 yo female	Case 1: Pemphigus flare Case 2: Dermatomyositis	Unknown. Case 3 ingested Aphanizomenon flos-aquae together with Spirulina.	Case 1: 7–10 days Case 2: 1–2 days	Case 1: A pemphigus flare was verified with a worse clinical picture that resolved after 2 weeks of stopping Spiruline and using prednisone. One week after this flare cleared, a second worse flare was observed. Case 2: erythema on the extensor arms, elbows, knuckles of the hands (Gottron’s sign), face, upper back, and neck. Positive ANA 1:160. Normal muscle enzymes. Skin biopsy: interface dermatitis and vacuolization. After 6 months: muscle weakness, CK 2018 U/L, aldolase 22.6 U/L.	Case 1: Spirulin stopping and prednisone. Case 2: Prednisone, hydroxychloroquine, tacrolimus ointment, and cetirizine.	Case 1: ND Case 2: ND

ANA: antinuclear antibodies; CK: creatine kinase; CT: computed tomography; IVIg: intravenous immunoglobulin; N: number; yo: years old

No article on positive effects of spirulina in autoimmune diseases was found.

It has been suggested that the immunostimulatory properties of Spirulina may lead to aberrant immune responses and subsequent autoimmunity [8]. In addition, chicks fed a Spirulina-supplemented diet exhibited a significant increase in macrophage numbers, along with an overall enhancement in phagocytic activity and nitrite production, both in LPS-induced and non-LPS-induced conditions [2]. Additionally, a human study demonstrated that oral administration of a extract of Spirulina enhanced NK cell function, as evidenced by increased interferon-γ production and cytolytic activity [3].

Future case reports on Spirulina and autoimmune disease onset or flare are awaited since this herbal supplement is consumed worldwide.

This review showed that spirulina ingestion may be correlated with dermatomyositis and pemphigus development/flare. Patients with autoimmune disease should be alert for flares after this herbal supplement ingestion. We concluded that Spirulina should be used with caution in patients with autoimmune diseases, especially dermatomyositis and pemphigus. Subjects using Spirulina should be monitored to the appearance of autoimmune conditions. Future studies on spirulina ingestion and its complications in autoimmune disorders are indeed needed.

## Electronic supplementary material

Below is the link to the electronic supplementary material.

Supplementary Material 1

## Data Availability

No datasets were generated or analysed during the current study.
