# Supplementary material for: Spirulina ingestion and autoimmune disease onset or flare
Source: Adv Rheumatol. 2025 Mar 15;65:15. doi: 10.1186/s42358-025-00446-7 (PMC13488625; doi:10.1186/s42358-025-00446-7)
Supplement: Supplementary file 1 [file 42358_2025_446_MOESM1_ESM.docx]

**Identification of studies via databases and registers**

Records removed *before screening*:

Duplicate records removed (n = 0 )

Records identified from*: 22

Databases (n = 4, Pubmed, Scopus, and Web of Science)

**Identification**

Records excluded**

(n = )

Review articles: 10

Experimental studies: 4

Records screened

(n = 22 )

Reports sought for retrieval

(n = 8)

Reports not retrieved

(n = 0)

**Screening**

Reports assessed for eligibility

(n = 4 )

Reports excluded: 0

Studies included in review

(n = 4)

**Included**
